# Laparoscopic versus open gastrectomy for elderly local advanced gastric cancer patients: study protocol of a phase II randomized controlled trial

**DOI:** 10.1186/s12885-018-5041-y

**Published:** 2018-11-16

**Authors:** Ziyu Li, Fei Shan, Xiangji Ying, Kan Xue, Jiafu Ji

**Affiliations:** Key laboratory of Carcinogenesis and Translational Research (Ministry of Education), Gastrointestinal Cancer Center, Peking University Cancer Hospital & Institute, No.52 Fucheng Road, Haidian District, Beijing, 100142 China

**Keywords:** Gastric cancer, Elderly, Laparoscopic gastrectomy, Open gastrectomy, Safety, Efficacy, Survival

## Abstract

**Background:**

Gastric cancer is one of the most common malignant tumors worldwide. With the rapid aging of global population, the number of elderly patients with local advanced gastric cancer is increasing. Surgery is the essential treatment for local advanced gastric cancer. However, elderly patients are at high risk of postoperative complications due to reduced functional reserve and increased comorbidities. Laparoscopic gastrectomy may be a promising surgery approach for elderly patients but its benefits remain controversial. We therefore proposed this randomized trial to evaluate the safety and efficacy of laparoscopic versus open gastrectomy for local advanced gastric cancer in patients aged 70 and above.

**Methods:**

The current study has a randomized, parallel controlled, single-center, open-label, superiority design with two arms. A sample of 180 local advanced gastric cancer patients aged 70 and above will be recruited in Peking University Cancer Hospital and Institute. Participants will be randomized to either receive open or laparoscopic gastrectomy. The primary outcome is surgical safety, including complication rate, reoperation rate, readmission rate, and mortality rate within 30 days after surgery. The secondary endpoints include postoperative rehabilitation status, one-year postoperative life quality, three-year overall and disease-free survival. Assessments will take place at baseline (before random assignment), at 30 days, one-year, and three-year after the surgery. The study has been approved by an ethical review board.

**Discussion:**

We hypothesized that laparoscopic gastrectomy is superior to open gastrectomy in terms of perioperative safety for local advanced gastric cancer patients aged 70 and above. If this hypothesis is statistically proved, the rational introduction of minimally invasive surgery technique in traditional gastrectomy can help improve the surgical safety for elderly patients, reduce patient financial burden, shorten hospital stay, and improve hospital beds turnover rate. Our research data will also provide high quality clinical evidence and data support for the conduction of multicenter phase III clinical trials.

**Trial registration:**

The study has been prospectively registered in ClinicalTrial.gov (NCT03564834).

## Background

Gastric cancer is one of the most common cancer and cause of cancer death in China and worldwide [1]. With the rapid aging of global population, the number of elderly patients with local advanced gastric cancer has been continuously increasing. Surgery is the essential treatment for local advanced gastric cancer. However, elderly patients are at high risk of postoperative complications due to reduced functional reserve and increased comorbidities. Studies have shown that elder patients can have postoperative complication incidence up to 18–32% and surgery-related mortality rate to 3.8–9.5% [2–4]. Therefore, elderly patients usually require more restrict operative injury control compared to the younger population. Surgical safety and effectiveness has become a crucial research focus for local advanced gastric cancer among elderly patients.

Laparoscopic gastrectomy is one of the standard treatments for early gastric cancer and has demonstrated its application value in local advanced gastric cancer [5–7]. Two recent meta-analysis on observational studies have shown the feasibility of laparoscopic gastrectomy in elderly gastric cancer patients [8, 9]. Compared to conventional open resections, elderly patients may benefit from the advantages of laparoscopic approach such as less trauma, less blood loss, faster bowel movement recovery, earlier food intake, and shorter hospitalization. However, laparoscopic gastrectomy raises issues such as prolonged operation time and disturbance of circulatory and respiratory dynamics by carbon dioxide pneumoperitoneum during the procedure [10]. Nonetheless, all currently available evidence comes from observational studies that are susceptible to bias and evidence on long-term survival is scarce. We therefore proposed to conduct this randomized controlled trial comparing the feasibility and survival benefit of laparoscopic with open gastrectomy for elderly patients with local advanced gastric cancer.

## Methods

### Objectives

The primary objective is to compare the perioperative safety of laparoscopic versus open gastrectomy for local advanced gastric cancer patients aged 70 and above. The secondary objective is to compare the surgical radicalness, postoperative recovery, one-year postoperative quality of life, three-year overall and disease-free survival of laparoscopic versus open gastrectomy for local advanced gastric cancer patients aged 70 and above.

### Hypothesis

Laparoscopic gastrectomy is superior to open gastrectomy in terms of perioperative safety for local advanced gastric cancer patients aged 70 and above.

### Study design, setting, and participant

**Fig. 1:**
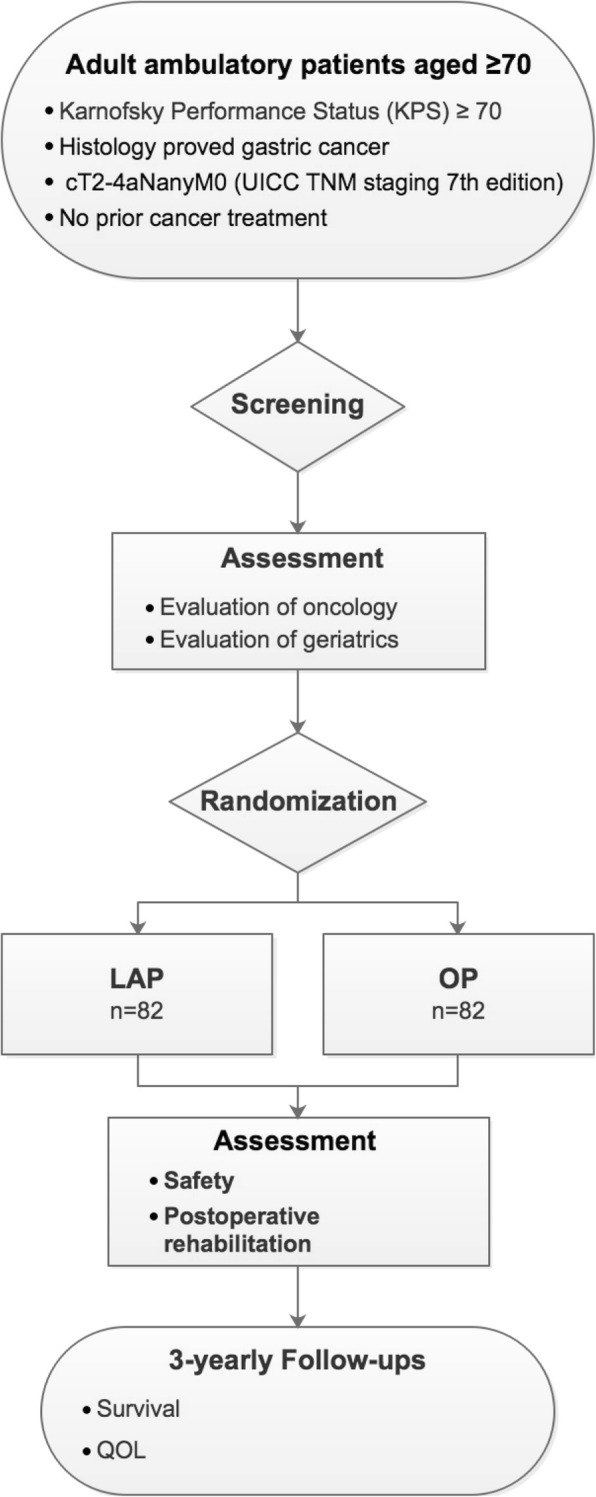
CONSORT diagram

This is a prospective, randomized, single-center, open-label phase II trial, which takes place in the Gastrointestinal Cancer Center of Peking University Cancer Hospital and Institute, China (Fig. 1). Adult ambulatory patients aged 70 and above, with Karnofsky Performance Status (KPS) ≥ 70, histology proved gastric cancer, cT2-4aNanyM0 (UICC TNM staging 7th edition), and no prior cancer treatment, will be screened for inclusion and exclusion criteria (Table 1) by the reception oncologist. Eligible patients will be invited for study participation at their first visit at the Gastrointestinal Cancer Center. The doctor who sees the patient will give a formal and detailed description of the study and its procedures. Upon the acquisition of patient written informed consent form, patients will undergo oncology evaluation (e.g. abdominal enhanced CT and/or MRI scan, chest plain scan CT, cervical lymph node, ultrasonography, pelvic ultrasonography/CT, serum tumor markers, laparoscopic exploration and free cytological examination of peritoneal cavity) as well as geriatric assessment with G8 screening tool. Patients will afterwards be randomized 1:1 to either laparoscopic or open surgery arm by the data manager in the team using random number table. None of patients, surgeons, or data analysts will be blinded.

**Table 1:** Patient eligibility — inclusion and exclusion criteria

Inclusion criteria	Exclusion criteria
• Ambulatory male or female aged 70 and above • Karnofsky score ≥ 70% • Histologically proven gastric adenocarcinoma in biopsy (including Lauren classification) Proven clinical stage of cT2-4aNanyM0 by baseline ultrasound endoscope, enhanced CT/MRI examination, or diagnostic laparoscopy using Habermann Standards • No past chemotherapy or radiotherapy before diagnosis • Primary tumor located at stomach, achievable naked-eye complete resection (R0/1) via distal subtotal or total gastrectomy plus lymphadenectomy • Haematology and biochemistry index meet the following: hemoglobin≥80 g/L, absolute neutrophils count (ANC) ≥ 1.5 × 109/L, platelet≥100 × 109/L, ALT、AST ≤ 2.5 times the upper limit of normal value, ALP ≤ 2.5 times the upper limit of normal value, serum total bilirubin< 1.5 times the upper limit of normal value, serum creatinine< 1 times the upper limit of normal value, serum albumin≥30 g/L • Heart and lung function can withstand surgery • No severe concomitant disease that leads to survival< 3 years • Willing and able to comply with study protocol • Written agreement consent before enrolment and full aware of the right to quit the study at any time with no loss	• Uncontrolled seizure, central nervous system diseases, or mental disorders; • Past history of upper abdominal surgery (except for laparoscopic cholecystectomy) • Past history of gastric surgery (including diagnosis procedure such as ESD and EMR) • Other malignant diseases in 5 years (except for cured skin carcinoma and cervical carcinoma in situ) • Clinical severe or active heart diseases, such as symptomatic coronary heart disease, NYHA grade II or above congestive heart failure, severe arrhythmia, or myocardial infarction in 6 months • Cerebral hemorrhage or infarction in 6 months • Organ transplant recipients under immunosuppressive therapy • Severe uncontrolled repeated infection or other severe uncontrolled concomitant diseases • Medium or severe renal damage (creatinine clearance rate ≤ 50 ml/min or serum creatinine> upper limit of normal value) • Other diseases requiring synchronous surgery • Requiring emergent surgery due to oncologic emergent (e.g. bleeding, perforation, obstruction) • FEV1 < 50% of expected value • Participated in other studies 4 weeks before the randomization.

The trial is funded by the Program for Healthcare Development Research & Technology, Beijing Municipal Health Bureau (2018–4-2156). This protocol and the informed consent forms have been reviewed and approved by Peking University Cancer Hospital and Institute Ethics Review Committee. We will obtain a new approval from the Committee if any amendments are made to the protocol or the informed consent form that may have an impact on the conduct of the study or potential benefit of the patient. The study has been registered in ClinicalTrial.gov (NCT03564834).

### Interventions

All patients will receive surgery within one week after randomization. Perioperative enteral/parenteral nutrition support will be allowed for patients with nutritional risk. The patients will be fasted of food and water 1 day and 4 h before the surgery, respectively. Patients will not normally receive preoperative gastrointestinal decompression, except for those with digestive tract obstruction. Prophylactic use of antibiotics during the perioperative period will be in accordance with relevant national regulations. All patients with pathology proved advanced gastric cancer will be suggested to use adjuvant chemotherapy, the regimen of which will be in line with the Japanese Gastric Cancer Treatment Guideline (version 5th) [11].

A standard laparoscopic or open radical gastrectomy will be performed by two experienced surgeons for all enrolled patients. In the open group, an approximately 20- to 25-cm incision will be made from the falciform process to the periumbilical area [12]. In the laparoscopic group, one 10-mm trocar for the camera will be inserted below the umbilicus [12]. Another three 10-mm ports will be inserted in the left upper quadrants 2 cm below left lower rib margins, the right and left flank areas, respectively [12]. One 5-mm trocar will be lastly placed at the right upper quadrants 2 cm below right lower rib margins [12]. Pneumoperitoneum will be achieved using carbon dioxide with a pressure maintained at 8-12 mmHg. Greater omentum resection, lymph node dissection, and vascular treatment will be performed under laparoscopy. Gastrectomy and digestive tract reconstruction may be performed by the surgeon under laparoscopy or assisted incision, as appropriate. All abdominal operation of laparoscopy will be videotaped. Anastomosis will be performed using the instrumental method. The specimen will be pulled out through a small median incision under the xiphoid (about 3–8 cm). In both groups, the range of gastric resection and lymph nodes dissections will be done in accordance with the Japanese Gastric Cancer Treatment Guideline (version 5th) [11]. Surgeons will decide the digestive tract reconstruction approach according to the intraoperative circumstances. Considering the healing capacity of elderly patients, local drainage will be placed on both sides of the anastomotic stoma.

For those receiving laparoscopic gastrectomy, the case will be required to be converted to open surgery if one of the following happens: confluent lymph nodes with long axis > 3 cm, severe or life-threatening intraoperative complications such as intra-abdominal massive haemorrhage, severe organ damage, or other technical or instrumental factors that require a conversion to open surgery.

### Assessment of outcomes

#### Primary endpoints

The primary endpoint is surgical safety, including complication rate, reoperation rate, readmission rate, and mortality rate within 30 days after surgery. Surgery related complications include incision complications (infection, effusion, dehiscence, poor healing), peritoneal effusion or abscess formation, hemorrhage (inside abdominal cavity, inside digestive tract), ileus, anastomotic leakage, anastomotic stenosis, intestinal fistula, lymphatic leakage, pancreatic fistula, gastroparesis, pancreatitis, lung infection, pleural effusion, urinary tract infection, renal failure, liver failure, cardio-cerebrovascular events (both lower extremities thrombosis, pulmonary embolism, myocardial infarction, arrhythmia, cerebral infarction, etc.), and others. Complications will be reported and graded according to the Clavien-Dindo classification of surgical complications.

#### Secondary endpoints

- Postoperative rehabilitation evaluation indicators: first time out of bed, first time of flatus/defecation, first time of semi-liquid diets, time of gastric tube removal, time of full removal of drainage, daily drainage volume, postoperative pain and the dosage of non-prophylactic analgesic drugs, postoperative blood transfusion volume, and postoperative hospitalization stay.
- One-year postoperative life quality: assessed using the European Organization for Research and Treatment of Cancer (EORTC) core 30-item Quality of Life Questionnaire for cancer patients (QLQ-C30) [13]. This questionnaire contains both Gastric (QLQ-STO22) [14] and Elderly Cancer Patients (ELD14) [15] modules and was developed to assess the generic and disease-specific quality of life of elderly gastric cancer patients. We will calculate summary scores from the mean of scales. Prior to calculating the mean, the symptom scales will need to be reversed to obtain a uniform direction of all scales. The summary score should only be calculated if all of the required scale scores are available (using scale scores based on the completed items, provided that at least 50% of the items in that scale have been completed.
- Three-year overall survival rate: overall survival is defined as the time interval from the time of the radical gastrectomy to the date of all-cause death or the last follow-up. Three-year overall survival rate will be calculated using the Kaplan-Meier Methods.
- Three-year disease-free survival rate: disease-free survival is defined as the time interval from the time of the radical gastrectomy to the date of the detection of cancer recurrence or the last follow-up. Three-year disease-free survival rate will be calculated using the Kaplan-Meier Methods.

### Data collection

Table 2 displays the schematic diagram for the timeline of patient assessment and data collection. Assessment on surgical safety and postoperative rehabilitation status will be performed by research physicians and recorded by the research nurse within 30-days after the gastrectomy. The case manager will collect demographic information at baseline, conduct life quality assessment one-year postoperative, and do follow-up on survival status till three-years after the last treatment. After gathering all relevant data collected by physicians, research nurse, case manager, as well as lab tests and imageological examinations, the data manager is responsible for data storage, security, management, and quality control. Data quality will be double-checked by the research physician aperiodically to further promote data accuracy and completeness.

**Table 2:** Schematic diagram for the schedule of enrolment, interventions, and assessments

TIMEPOINT	STUDY PERIOD						
	Enrolment	Allocation	Post-allocation				Close-out
	*-2 to − 4 weeks*	0	*1 week*	*4 weeks*	*Year 1* ^a^	*Year 2* ^a^	*Year 3* ^b^
ENROLMENT:							
Eligibility screen	X						
Informed consent	X						
Allocation		X					
INTERVENTIONS:							
*Open gastrectomy*			X				
*Laparoscopic gastrectomy*			X				
ASSESSMENTS:							
*Physical examination*	X				X	X	X
*Laboratory tests*	X				X	X	X
*Oncology assessment*	X				X^c^	X^c^	X^c^
*Geriatric assessment*	X						
*Surgical safety*				X			
*Postoperative rehabilitation*				X			
*Life quality*					X^d^		
*Survival status*					X	X	X

^a^conducted on a 3-month basis

^b^conducted on a 6-month basis

^c^conducted if recurrence is suspected

^d^conducted at the end of Year 1

### Patient follow-up

Information on prognostic status is collected via follow-up till 3 years after the last treatment. Follow-up will be conducted on a 3-month basis in the first two years and every half-year in the third year. In each follow-up, participants receive physical examinations (i.e. height, weight, KPS, routine examination of heart/lung/abdomen), laboratory tests (including blood cell test, blood biochemical test, and serum tumor marker test), imageological examinations (including ultrasonography [every 3 months], enhanced CT/MRI [every 6 months], endoscopy [every 12 months], and chest radiograph). Tumor assessment will be conducted if recurrence is suspected, and further treatment such as surgery or chemotherapy will be administered when needed and will be recorded in the case report form. To promote participant retention and complete follow-up, three attempts will be made to contact and remind the participants to come to the hospital for follow-up, and their transportation fee will be covered by the research project.

### Statistical analysis

On the basis of prior research, the conventional open surgery group is expected to have a 30-day postoperative complication rate of 35%. After the repeated discussion among investigators, a consensus was reached that a 19% decrease in the complication rate was considered as superiority of laparoscopic gastrectomy over conventional open approach. PASS software returned a sample size of 164 (82 per arm) is planned, with a type I error of 0.05 (two-sided) and a statistical power of 80%. The total sample size needed is 180 (90 per am) after taking into account of a 10% dropout rate.

Data analysis will be performed on both intention-to-treat and per-protocol basis. Outcome data obtained from all participants will be included in the intention-to-treat analysis, regardless of protocol adherence; whereas in the per-protocol analysis, data of non-adherers will be excluded. For variables with a significant amount of missingness, multiple imputations will be conducted for the purpose of sensitivity analysis. Of the clinical and pathological characteristics, categorical data will be presented as number and percentage and continuous variables as mean and standard deviation if normally distributed or as median and range if otherwise. The primary analysis in this study aims to compare the between-group difference in 30-days postoperative complication rate, reoperation rate, readmission rate, and mortality rate. Pearson’s Chi-squared tests or Fisher’s Exact tests, as appropriate, will be performed for this purpose. For the continuous variables of secondary outcomes, this will be done by independent samples t-tests or Mann-Whitney U tests, as appropriate. The overall survival curves will be constructed as time-to-event plots using the Kaplan–Meier method. Log-rank tests will be used to make a simple comparison of the survival curves. Disease-free survival will be analyzed in the same manner. Proportional hazard Cox regression adjusted for variables will be employed when necessary. Subgroup analyses will be conducted by G8 score, age group, TNM stage, tumor location, laparoscopy-assisted or total laparoscopic gastrectomy, distal or total gastrectomy, and comorbidities status. All statistical analyses will be conducted in standard statistical software such as SAS and STATA with a significance level of 0.05 (two-sided).

### Data monitoring, auditing, and interim analysis

Data monitoring and auditing will be conducted by the funding agency annually. An interim-analysis will be performed by an independent statistician when half of the patients have been randomized. The trial will be stopped if one treatment is found to be statistically more beneficial or harmful than the other.

### Adverse events

Adverse events are any unfavourable or unintended events that affect patients on study, regardless of the relevance to the treatment [16]. Any adverse events will be recorded in detail on the CRF regarding its occurrence time, duration, relevance to the treatment, stopping or continuing of the treatment, etc. [16]. Events are defined as serious adverse events if leading to death, prolongation of hospitalization, permanent or severe disability, teratogenesis or carcinagensis, and significant clinical sequela [16]. The occurrence of serious adverse events will be reported to Peking University Cancer Hospital Ethics Committee within 24 h of the initial discovery [16].

## Discussion

Old age is recognized as a risk factor for surgery. Surgeons tend to be cautious and conservative in choosing treatment options for elderly patients with gastric cancer: often a more secure but less radical surgical approach is preferred [17]. However, thanks to the continuous improvement of modern surgical technology and perioperative nursing standards, elderly patients can now often better tolerate large operation and age is no longer the factor restricting the choice of surgical approach [18–21]. The change of this concept also makes laparoscopy as the most rapid developing minimally invasive technique used in gastrointestinal tumor surgery, and its application in the elderly patients is increasing. A number of studies have shown that laparoscopic gastrectomy can result in smaller incision, less bleeding, alleviated pain, and decreased surgical stress, therefore should theoretically have a protective effect for the elderly patients [22–25].

However, laparoscopic surgery requires the establishment of artificial pneumoperitoneum, which may cause the shrink of thoracic cavity volume and the decrease of lung compliance during the surgery. Additionally, when the abdominal cavity is in high abdominal pressure state for a relatively long time, the reflux of visceral and body cavity vein can be affected. This will consequently cause the damage to liver function and small intestine mucosal barrier, the stasis of lower limb vein blood flow, resulting in an increased risk of postoperative infection and thrombosis. Moreover, the CO_2_ used to form the pneumoperitoneum can be absorbed into the blood through peritoneal, and cause high blood carbonate and acidosis if not being fully compensated. The impact of this process on health is generally very limited. However, when the patient is at advanced age with cardiac and respiratory comorbidities, it is more likely to occur and make a negative impact on the status of the whole body if not being corrected in time [26].

Therefore, the benefits of laparoscopic surgery for the elderly patients with gastric cancer remain controversial. Our team’s previous meta-analysis likewise found that although available evidence suggests the feasibility and potential of laparoscopic surgery for elderly patients with advanced gastric cancer, these studies suffer from pitfalls such as the inconsistency on definition of advanced age, surgical quality measurement, complication assessment standards, and publication bias [8]. There is still a lack of high-level clinical evidence from randomized controlled studies.

The current proposed randomized trial therefore aims to evaluate the safety and efficacy of laparoscopic radical gastrectomy for local advanced gastric cancer elderly patients. If our research hypothesis is statistically proved, the rational introduction of minimally invasive surgery technique in traditional tumor surgery can help improve the surgical safety for the elderly patients with gastric cancer, shorten hospital stay, reduce patient financial burden, improve the turnover rate of hospital beds, and promote the economic benefits of the hospital. Our research data will also provide high quality clinical evidence and data support for the conduction of multicenter phase III clinical trials.

## Appendix

### Other supplementary information

**Protocol Version**

Issue Date: March 22, 2018

Protocol Amendment Number: 01

Author(s): Fei Shan,MD; Xiangji Ying, MPH

**Sponsor Contact Information**

Trial Sponsor: Beijing Municipal Commission of Health and Family Planning

Sponsor's Reference: Program for Healthcare Development Research & Technology (2018-4-2156)

Address: Tower B, Zhonghuan Office Building, No. 70 Zaolin Qian Street, Xicheng District, Beijing 100053, China

Telephone: +86-10-83970601

**Roles and responsibilities**

***Principal investigator and research physicians***

Design and conduct of the trial

Preparation of protocol and revisions

Preparation of case report forms

Preparation of materials for international review, board/independent ethics committee applications

Patient enrolment

Performing surgery

Publication of study reports

Members of Trial Management Committee

Annual progress reporting to the funder and ethics committee

Serious adverse events reporting to ethics committee

Responsible for trial data file

Budget administration

Data verification

***Data manager***

Randomization

Maintenance of trial IT system and data entry

Patient follow-up

Data verification

***Case manager and research nurse***

Data collection and patient follow-up

## Abbreviations

EORTC: European Organization for Research and Treatment of Cancer
KPS: Karnofsky Performance Status
QLQ: Quality of Life Questionnaire
TNM: Tumor, node, metastasis
UICC: Union for International Cancer Control
